# HIV-Associated Cryptococcal Disease in Resource-Limited Settings: A Case for “*Prevention Is Better Than Cure*”?

**DOI:** 10.3390/jof3040067

**Published:** 2017-12-02

**Authors:** Rita O. Oladele, Felix Bongomin, Sara Gago, David W. Denning

**Affiliations:** 1 Division of Infection, Immunity and Respiratory Medicine, Faculty of Biology, Medicine and Health, The University of Manchester, Manchester M13 9PL, UK; drritaoladele@yahoo.com (R.O.O.); sara.gago-2@manchester.ac.uk (S.G.); ddenning@manchester.ac.uk (D.W.D.); 2 Department of Microbiology and Parasitology, College of Medicine, University of Lagos, Lagos ,P.O.Box 132, Nigeria; 3 Global Action Fund for Fungal Infections, 1211 Geneva 1, Switzerland; 4 The National Aspergillosis Center, Education and Research Centre, Wythenshawe Hospital, Manchester University NHS Foundation Trust, Manchester M23 9LT, UK; 5 Manchester Fungal Infection Group, Core Technology Facility, The University of Manchester, Manchester M13 9PL, UK

**Keywords:** cryptococcal disease, resource-limited settings, cryptococcal polysaccharide capsular antigen (CrAg) test, prevention and treatment

## Abstract

Cryptococcal disease remains a significant source of global morbidity and mortality for people living with HIV, especially in resource-limited settings. The recently updated estimate of cryptococcal disease revealed a global incidence of 223,100 cases annually with 73% of these cases being diagnosed in sub-Saharan Africa. Furthermore, 75% of the estimated 181,100 deaths associated with cryptococcal disease occur in sub-Saharan Africa. Point-of-care diagnostic assays have revolutionised the diagnosis of this deadly opportunistic infection. The theory of asymptomatic cryptococcal antigenaemia as a forerunner to symptomatic meningitis and death has been conclusively proven. Thus, cryptococcal antigenaemia screening coupled with pre-emptive antifungal therapy has been demonstrated as a cost-effective strategy with survival benefits and has been incorporated into HIV national guidelines in several countries. However, this is yet to be implemented in a number of other high HIV burden countries. Flucytosine-based combination therapy during the induction phase is associated with improved survival, faster cerebrospinal fluid sterilisation and fewer relapses. Flucytosine, however, is unavailable in many parts of the world. Studies are ongoing on the efficacy of shorter regimens of amphotericin B. Early diagnosis, proactive antifungal therapy with concurrent management of raised intracranial pressure creates the potential to markedly reduce mortality associated with this disease.

## 1. Introduction

The pathogenic encapsulated yeasts in the genus *Cryptococcus* remains one of the most important opportunistic fungal pathogens worldwide [1]. Cryptococcosis is associated with very high morbidity and mortality both in immunocompetent and immunocompromised patients [2]. *Cryptococcus neoformans,* which predominantly affects immunocompromised patients, and *Cryptococcus gattii*, which can infect both immunocompetent and immunocompromised individuals, are the two major *Cryptococcus* species causing human disease of the more than 30 species ubiquitously distributed in the environment [3]. Disseminated *Cryptococcus neoformans* infection is largely seen in individuals with advanced HIV infection (CD4 < 200 cells/µL); it is an AIDS defining disease accounting for up 15% of AIDS-related death globally and even higher (20–50%) in resource limited settings [4,5,6,7].

## 2. Epidemiology of Cryptococcal Disease

Until the late 1970s and early 1980s, cryptococcal meningitis was an uncommon infection. However, the advent of the HIV/AIDS pandemic, an increase in the number of persons with malignancies (and their therapeutic modalities) and solid organ transplant recipients led to the rise in the incidence of this fatal opportunistic mycosis [8,9,10,11]. In fact, in the mid-1980s, cryptococcosis in AIDS accounted for more than 80% of the total cases worldwide. Although the incidence of HIV-associated cryptococcal infection has reduced significantly in industrialised countries with the widespread implementation of successful anti-retroviral therapy (ART) programs, the incidence of cryptococcal meningitis is still high, especially in sub-Saharan Africa and some parts of Asia [12]. Treatment of Cryptococcosis is still below standard in most low resource settings, given limited access to ART, the poor availability of first-line antifungal drugs used in the treatment of cryptococcal meningitis and muted uptake of recommendations for management of the increased intracranial pressure. The cryptococcal meningitis case fatality rate was predicted as 35–65% in HIV-infected African patients compared to 14–26% amongst HIV-infected patients living in industrialised countries [13,14,15,16]. However, recent works have demonstrated these numbers to be underestimated, and the cryptococcal meningitis one-year mortality in low-income countries would be higher than 70% (range: 50–81%) for those in care and 100% for those not in care [6]. A number of challenges exists in the management of cryptococcal meningitis in most of the low- and middle-income countries (LMICs); (1) diagnostic resources are limited, (2) there are no clinical criteria that consistently predict the diagnosis of cryptococcal meningitis, (3) not all patients receive fluconazole monotherapy in the suppression phase, and (4) lumbar punctures for both diagnostic and therapeutic indications [6,17].

Over the last three decades of the HIV pandemic, extensive research has gone into in improving cryptococcal meningitis management, which has resulted in the revision of international guidelines focusing on the diagnosis and management of the disease, however, the actual implementation of these guidelines has been varied in different nations to suit their epidemiological picture [18]. In 2011, the World Health Organization (WHO) and the Centre for Disease Prevention and Control (CDC) recommended cryptococcal antigenaemia screening for people living with HIV with CD4 counts < 100 cells/µL and who are not on ART [19,20]. HIV national guidelines in some low resource settings such as Botswana, Kenya, Mozambique, Namibia, Rwanda, South Africa, Uganda and Zimbabwe have incorporated pre-emptive antifungal therapy in patients with a positive cryptococcal antigen test [6,7,21]. However, in some African countries such as Botswana or South Africa, the burden of cryptococcal meningitis has increased in the last years in spite of the access to ART. In fact, in Cape Town, South Africa, 20% of HIV-infected patients presenting with cryptococcal meningitis were already receiving ART (after a median duration of 41 days) with 29% mortality [22].

The global burden of cryptococcal meningitis has been recently re-estimated at 223,100 cases (162,500 cases in sub-Saharan Africa) leading to 181,100 annual deaths (135,900 deaths in sub-Saharan Africa) [6]. The highest annual incidence of cryptococcal meningitis has been found in Nigeria (27,100 cases), South Africa (21,400 cases), Mozambique (18,600 cases), India (18,300 cases), Uganda (12,200 cases), Ethiopia (9600 cases), Kenya (9000 cases) and Zambia (5000 cases). Estimations were performed based on cryptococcal polysaccharide capsular antigen (CrAg) prevalence across 46 studies, most of them in LMIC settings. The average global CrAg prevalence in HIV-infected patients with CD4 < 100 cells/μL stands at 6.0%; most of the countries in resource limited settings have a prevalence well above this value [6]. The highest CrAg prevalence was reported in the Democratic Republic of Congo (15.2%) [23], Cambodia (10.8%) [24], Thailand (12.9%) [25], Nigeria (12.7%) [26] & 8.9% [27], and Ethiopia (11.2%) [28]. Although the study from Rajasingham et al. [6] has represented a huge advance in determining the global burden of cryptococcal meningitis across the world, these figures might still be underestimated due to the heterogeneity in the populations within the CrAg seroprevalence publications including new HIV patients, ART naïve patients or HIV patients on ART with different HIV severities.

## 3. Cryptococcal Disease Diagnosis in Low Resource Settings

Early diagnosis of cryptococcal infection is the key to improving outcomes. Traditionally, cryptococcal infection has been diagnosed by India ink microscopy on cerebrospinal fluid (CSF), culture or latex agglutination for cryptococcal antigenaemia.

### 3.1. Conventional Methods

Cryptococcal meningitis diagnosis can be achieved by directly examining the CSF for the presence of the yeast using India ink stain, this method has a low sensitivity of 50–70% when yeast burdens are higher than 10^3^ CFU/mL [20]. The sensitivity is even lower in the early stages of the disease or when patients are on antifungal therapy [29]. These limitations contribute to misdiagnosis, thus increasing the burden and mortality of the disease [2]. Some inexperienced microscopists may mistake yeast cells for lymphocytes, especially if the yeast cells have a thin capsule.

Mycological culture of CSF samples is considered the “gold standard” diagnostic method. It is usually positive at 48–72 h for antifungal naïve patients or longer (up to four weeks) for patients on antifungal therapy. *Cryptococcus* species is isolated in 75–90% of CSF samples of meningitis cases and ~35–70% blood cultures [20]. However, is more reliable with larger quantity specimens, laboratory infrastructure, skilled personnel and delays in obtaining a result make culture clinically unhelpful for initial management decisions. Moreover, in patients with cryptococcal meningitis and immune reconstitution syndrome, fungal burdens can be very low leading to a false negative culture result [30]. A quantitative culture of defined volumes of CSF has been of great value in assessing the value and rapidity of fungal killing by different antifungal regimens. Culture also allows the detection of resistance, which is problematic with fluconazole.

### 3.2. Immunodiagnosis of Cryptococcosis 

Cryptococcal polysaccharide capsular antigen (CrAg) is shed into the bloodstream very early in the dissemination of cryptococcal disease [31]. Detection of CrAg in serum and CSF by latex agglutination has been extensively utilized in the last 40 years with sensitivity and specificity values ranging from 93–100% and 93–98%, respectively [2]. CrAg is measurable in serum between 5 and 234 (median 22) days before the onset of symptoms of cryptococcal meningitis, thus making screening for serum CrAg and subsequently the treatment of those with a positive test result a conceivable means of lowering cryptococcal-meningitis-associated mortality [31]. Until the last decade, CrAg tests were performed by latex agglutination assays (LA) or enzyme immunoassay (EIA). However, these methods require refrigeration, a cold chain for specimen transport, and technical expertise. When patients have high titers, they are also expensive, requiring multiple sample dilutions and assays. They are often performed only in reference/diagnostic laboratories far removed from patients, potentially limiting their clinical utility and in addition, they are expensive [32]. 

In July 2011, the US Food and Drug Administration approved the use of a newly developed rapid point-of-care lateral flow assay (LFA) (IMMY, Inc., Norman, OK, USA) for the diagnosis of cryptococcal meningitis. This test uses an immunochromatographic test strip that contains gold-conjugated monoclonal antibodies which bind to glucuronoxylomannan (GXM) cryptococcal antigen from all cryptococcal serotypes. Other LFA tests are also available from Biosynex and Dynamiker. The LFA assay has a number of qualities that make it perfect for use in resource-limited settings; it is cheap (approx. $4 per test) [33], has a high sensitivity/specificity, allows point-of-care or laboratory testing, can be used on a fingerprick blood sample, has a rapid turnaround time, does not requires electricity (a major challenge in sub-Saharan Africa), the diluent and test strips are stable at room temperature with a long shelf life (up to two years), it is easy to perform (minimal training required), and there is no need for processing of samples (e.g., pre-treatment, heat inactivation) or specialized laboratory equipment [34,35]. Additionally, some publications have revealed the utility of this LFA as a quantitative test [36].

The specificity and sensitivity of the CrAg LFA test in blood samples ranges from 99.6 to 100% and 92–100% respectively [34,37,38,39] and is comparable with results from other antigen-based tests [37,40,41,42]. A study from Tanzania demonstrated a 100% agreement between serum LFA and LA in evaluating CrAg prevalence in asymptomatic, ART-naïve patients; thus supporting LFA as a good substitute to LA assay for use in CrAg screening [42]. A publication from a study in Africa demonstrated that there is 100% agreement between whole blood, serum, and plasma CrAg LFA testing, signifying that finger prick is a feasible alternative for point of care testing of CrAg, especially in the absence of a phlebotomist [43]. Systematic review of LFA studies, revealed a median CSF sensitivity of 100%, and a median specificity of 97.7% [44]. However, published data demonstrates poor performance of LFA with urine (has good sensitivity but poor specificity) and saliva samples (excellent specificity but poor sensitivity) [34,37,38,39,45]. A positive CrAg test has also been shown to be useful as a predictor of cryptococcal meningitis and mortality after ART initiation [31,46,47].

## 4. Management of Cryptococcal Meningitis in Resource-Limited Settings

**Figure 1:**
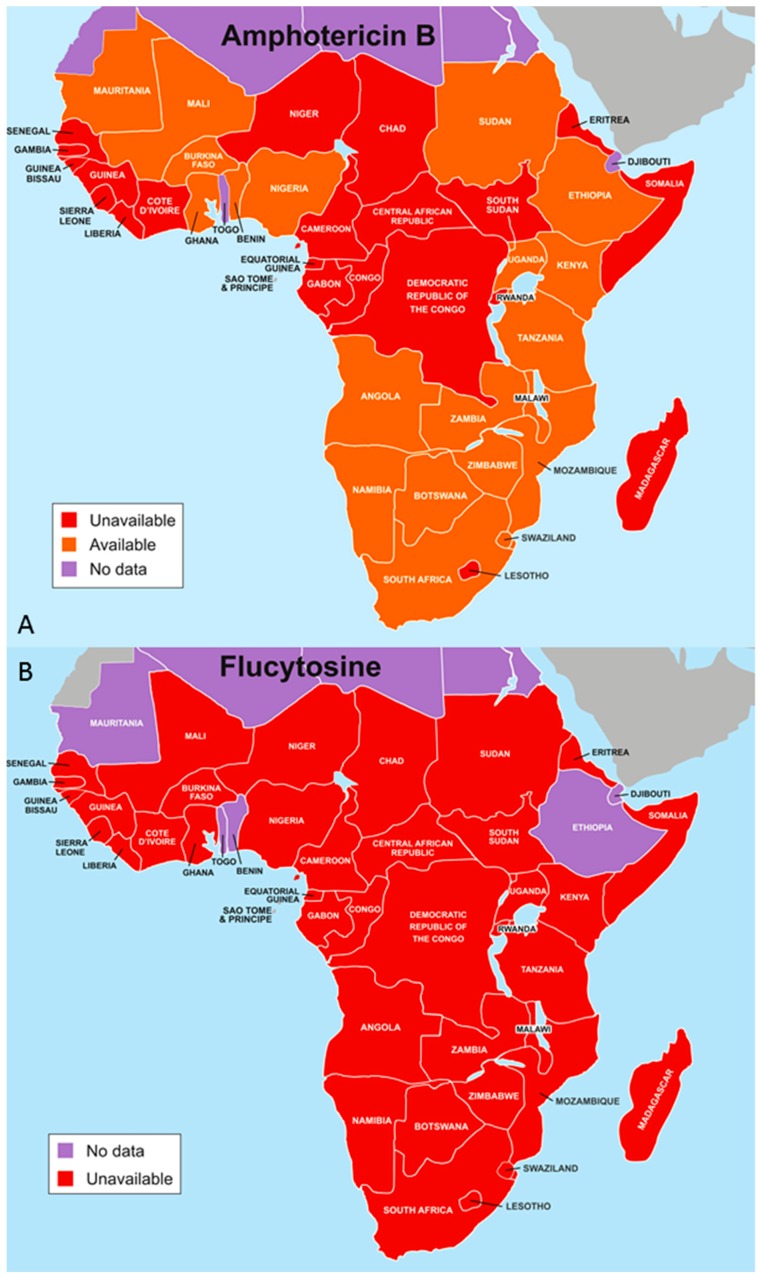
(**A**) Availability of amphotericin B in sub-Saharan Africa; (**B**) Lack of availability of flucytosine in sub-Saharan Africa (maps courtesy of Global Action Fund for Fungal Infections, GAFFI).

The management of cryptococcal meningitis remains a challenge in resource-limited settings, this is mainly due to the fact that patients often present late in care with advanced disease, ill-equipped health facilities for the diagnosis and management of cryptococcal meningitis and its complications, and the non-availability of essential antifungal agents (Figure 1) [48]. It is clear that a good understanding of the management of cryptococcal disease is associated with better patient outcomes. The key elements in the management of acute cryptococcal meningitis includes optimal phased-antifungal therapy, recognition and treatment of raised intracranial pressure, early detection and management of cryptococcal immune reconstitution inflammatory syndrome (C-IRIS), and the use of lipid formulation of amphotericin B in individuals with impaired baseline renal function or anaemia [49] (Table 1).

### 4.1. Optimal Antifungal Therapy

Induction therapy with amphotericin B plus flucytosine is associated with improved survival among patients with cryptococcal meningitis, as compared to amphotericin B alone or amphotericin B plus fluconazole [50]. However, flucytosine is not widely available in resource-limited settings [51], as such the use of amphotericin B deoxycholate monotherapy or amphotericin B deoxycholate in combination with fluconazole or high dose fluconazole monotherapy are feasible and common treatment options in resource-limited settings [2,48,52,53]. Fluconazole is fungicidal at a dose of 1200 mg/day and it is fungistatic when administered at a dose of 800 mg/day [54,55]. The survival rate on these regimens at 12 weeks is only about 30–60% compared to 80–90% in patients who received amphotericin B-flucytosine combination therapy [50,56]. The use of flucytosine in combination with fluconazole is associated with additive toxicities [57]. A systematic review of cryptococcal treatment trials in resource-limited areas showed a cost benefit in using short-course (seven days) amphotericin B induction therapy coupled with high-dose (1200 mg/day) fluconazole [58].

Lipid formulations of amphotericin B (i.e., liposomal amphotericin B 3–4 mg/kg/d up to a maximum dose of 6 mg/kg/d) can be used in place of conventional amphotericin B and are less nephrotoxic, although not more effective. They are preferred for cryptococcal meningitis in patients who are immunosuppressed through organ transplantation, who do not tolerate conventional amphotericin B well. Amphotericin B-based induction is often prolonged beyond two weeks in these cases, and in the non-HIV, non-transplant patient group, including those who are immune-competent and those infected with *C. gattii*. Recent data from a phase II randomized controlled non-inferiority trial from Tanzania showed that single dose 10 mg/kg of liposomal amphotericin B is well-tolerated with a non-inferior early fungicidal activity (EFA) compared to 14-day courses of 3 mg/kg liposomal amphotericin B in the treatment of HIV-associated cryptococcal meningitis [59]. The induction phase is followed by a consolidation phase and long-term suppressive antifungal therapy using fluconazole monotherapy [49]. Long-term antifungal suppression is required until immune reconstitution to reduce the high possibility of relapse for patients who receive induction therapy only [60].

In search for a novel agents for treating cryptococcal meningitis, a recent study in Tanzania has shown that the antidepressant drug sertraline in combination with fluconazole improves the two-week CSF fungal clearance rate and clinical outcomes and is superior to fluconazole monotherapy or short course amphotericin B therapy [61].

### 4.2. Antiretroviral Therapy Timing after Initiation of Antifungal Therapy

The Infectious Diseases Society of America (IDSA) clinical guideline for the management of cryptococcal meningitis recommends initiation of ART 2–10 weeks after the commencement of the initial antifungal treatment, based on earlier studies [49]. However, the cryptococcal optimal ART timing (COAT) trial conducted in Uganda and published in 2014 showed that earlier (1–2 weeks) ART initiation in cryptococcal meningitis results in higher mortality compared with deferred (five weeks or more) ART initiation [62]. The increased mortality from early ART in the COAT trial was immunologically mediated, as a follow-up study on the cryopreserved CSF and serum of these patients showed increased CSF pleocytosis, microglial activation, and T-helper 2 responses within the central nervous system [63]. It is thus generally accepted now that ART initiation should be delayed for at least four weeks following induction antifungal therapy, in order to prevent other HIV-related complications without exacerbating immune reconstitution reactions. Initiation of antiretroviral therapy at the time of diagnosis of cryptococcal meningitis increases mortality [62].

### 4.3. Management of Intracranial Pressure

**Figure 2:**
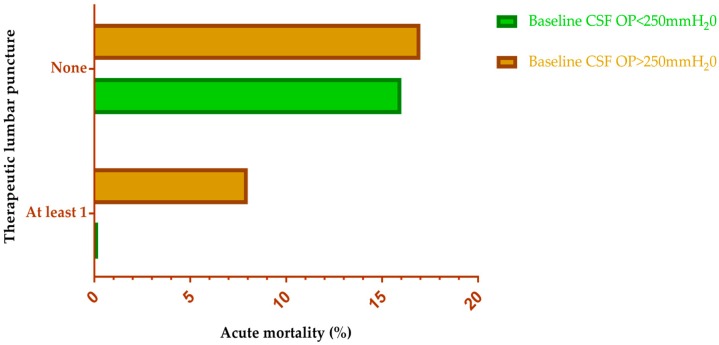
Therapeutic lumbar puncture and acute mortality in HIV-infected individuals with cryptococcal meningitis. Repeated therapeutic lumbar puncture is associated with improved survival regardless of initial CSF opening pressure. Data obtained from Rolfes et al. [67]. CSF: cerebrospinal fluid. OP: opening pressure

Raised intracranial pressure (ICP) is very common, occurring in up to over 60% of patients with cryptococcal meningitis [15] and is associated with reduced short-term survival and impaired treatment response [64]. Early recognition of raised ICP followed by aggressive ICP reduction by means of repeated therapeutic lumbar (LP) drainage is associated with better outcomes [65]. The mechanism of raised ICP is poorly understood, but is thought to be due to the direct obstruction of arachnoid villi by cryptococcal yeast cells [66]. In a nutshell, the aggressive management of increased ICP is as important as antifungal therapy in the management of cryptococcal meningitis. A clinical trial done in Uganda and South Africa to determine the effect of therapeutic LP on acute mortality from cryptococcal meningitis between individuals receiving at least one therapeutic LP with individuals not receiving therapeutic LPs, showed a 69% improvement in survival regardless of initial CSF opening pressure [67] (Figure 2). Though rare, there are very few complications such as brain herniation, subarachnoid haemorrhage and haematoma, bacterial meningitis and sepsis that complicate LP, especially repeated LP [68]. Contraindications for LP include local sepsis on the back, ongoing anti-coagulation therapy and focal neurological deficits suggestive of a cerebral space-occupying lesion (SOL). A head CT scan is required to rule out SOL.

In resource-limited settings, the tools for repeated lumbar punctures and manometry are not available, or they exist in such limited supply as to impede the provision of optimal care for the majority of persons with HIV-associated cryptococcal meningitis. In addition to these economic barriers, there are also cultural barriers to care; for example, permission for additional lumbar punctures for the management of increased intracranial pressure is often denied by patients or their families due to misunderstanding and fear [68].

Occasional patients have persistently raised CSF pressure, despite three or four lumbar punctures and good antifungal therapy. The best option for most of these patients is the insertion of a lumbar drain. An alternative is the insertion of a ventricular drain, either directly draining externally or a ventriculo-peritoneal shunt [66,69].

Other measures to reduce raised CSF pressure are ineffective or harmful. The controversial role of corticosteroid in the management of cryptococcal meningitis has recently been laid to rest. A double-blind, randomized, placebo-controlled trial, that recruited 451 adult patients with HIV-associated cryptococcal meningitis in Vietnam, Thailand, Indonesia, Laos, Uganda, and Malawi did not show any survival benefit in using adjunctive dexamethasone, but rather adverse events and disability in patients that received it [70].

### 4.4. Treatment Outcomes

#### 4.4.1. Mortality

Cryptococcal meningitis accounts for up to 15% of HIV-related deaths [6]. It is one leading causes of early mortality among HIV-infected adults in sub-Saharan Africa [71]. Without treatment, the two-week mortality associated with acute cryptococcal meningitis is almost always 100% [72]. The early mortality associated with acute cryptococcal meningoencephalitis in the developed countries with access to ART is as low as 10–20% when managed with combination antifungal therapy. In patients treated late, with fluconazole monotherapy, the outcome is much worse, with over 50% mortality at 10 weeks [15,73,74]. Tenforde and colleagues comprehensively reviewed the published mortality rates attributed to cryptococcal meningitis in resource-limited settings [75]. In their review, the ten-week mortality with amphotericin-based treatment regimens ranged between 22% and 36% and the twelve-month mortality between 41% and 56% [75]. A more recent report of a cohort study from Botswana enrolling 236 individuals with HIV-associated cryptococcal meningitis showed an overall mortality of 62%. The two-week, 10-week and one-year mortality were 26%, 50%, and 65%, respectively [76].

#### 4.4.2. Relapses and Persistent Cryptococcal Disease

Without consolidation and maintenance antifungal therapy, recurrence is very common occurring in up to 40–50% of the patients after a successful induction antifungal therapy [60]. The diagnosis of persistence and relapse is based on CSF cultures and not on biochemical markers, microscopy or antigen tests. Management involves re-initiation of the induction agents at a higher dose and longer duration (≥4 weeks). The determination of the antifungal susceptibility profile of the relapse/persistent isolates is crucial as this might indicate antifungal drug resistance. Adjunctive interferon gamma supplementation may be considered in selected patient groups [77]. Both relapsed and persistent cryptococcal disease should be clearly differentiated from C-IRIS, as the later may require steroid therapy without alteration/re-initiation of induction antifungal agents [49]. The best predictors of recurrence-free survival are fluconazole treatment, a lower serum cryptococcal-antigen titre, and more prolonged primary therapy with flucytosine [60]. Maintenance therapy with fluconazole is highly effective in preventing recurrent cryptococcal infection, and it remains the treatment of choice for maintenance therapy for AIDS-associated cryptococcal disease. Flucytosine may contribute to the prevention of relapse if used during the first two weeks of primary therapy [60,78]. Immune restoration and low serum cryptococcal antigen titres are associated with lower cryptococcosis relapse rates [79].

#### 4.4.3. Cryptococcal Immune Reconstitution Inflammatory Syndrome (C-IRIS)

C-IRIS is an emerging problem in resource-limited settings. It can present any time after initiation of ART and can present as late as two years, and this can be explained from the persistence of CrAg in the systemic circulation [80,81]. The exact burden of C-IRIS is unclear in these settings, it could be because of the difficulties in diagnosis and lack of clarity in the diagnostic criteria [81]. C-IRIS usually presents with central nervous system manifestations, but it can also display pulmonary disease and/or lymphadenitis. Meningitis with C-IRIS is usually characterized by a higher cerebrospinal fluid (CSF) white blood cell count and a lower cryptococcal antigen titre compared with the typical clinical picture for cryptococcal meningitis, C-IRIS can be potentially life threatening and is associated with increased mortality [82]. The management of IRIS with corticosteroids may increase the risk of culture-positive cryptococcal meningitis relapse, which may further increase the risk of recurrent C-IRIS and resulting complications including death.

#### 4.4.4. Sequelae of Cryptococcal Meningitis

Visual loss is the major complication of sustained high ICP in cryptococcal meningitis [66]. It has been documented in both in both AIDS and non-AIDS patients [66]. Other complications include hearing loss, strabismus, diplopia in association with reduced acuity; and marked papilledema [66,69]. A report from Baltimore, revealed that nine of 27 AIDS patients with cryptococcal meningitis developed neuro-ophthalmic lesions, mostly cranial nerve palsies; three patients developed papilloedema, and one an optic neuropathy [83]. Moreover, Bicanic and colleagues demonstrated that aggressive management of raised ICP through repeated CSF drainage appeared to prevent any adverse impact of raised opening pressure on outcome in patients with cryptococcal meningitis [84].

## 5. Antifungal Prophylaxis for Cryptococcal Meningitis

**Figure 3:**
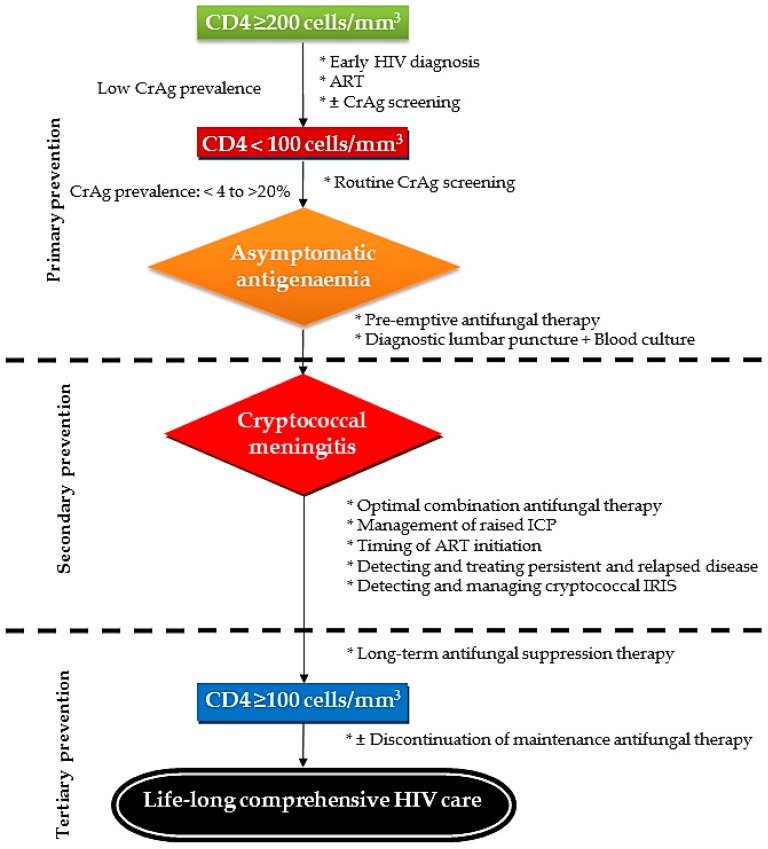
Levels of prevention for cryptococcal disease stratified by immunological status. CD: Cluster of differentiation. IRIS: Immune reconstitution inflammatory syndrome. ICP: Intracranial pressure. ART: Antiretroviral therapy.

Antifungal prophylaxis is one of the key components of the comprehensive care for HIV-infected persons who are at risk of developing cryptococcal meningitis or patients who have had a recent episode of cryptococcal disease (Figure 3).

### 5.1. Primary Prophylaxis

In the early 2000s, a multicenter, randomized, double-blind, placebo-controlled trial of fluconazole for primary cryptococcal meningitis prophylaxis in HIV-infected patients with a CD4+ T-cell count < 100 cells/µL showed that patients in the placebo group were 4.3 times more likely to die than those in the fluconazole group [85]. This is consistent with similar findings from Uganda [86]. In low-resource settings where antiretroviral therapy is not universally available and the burden of cryptococcal meningitis is very high, the WHO has recommended primary fluconazole prophylaxis for all HIV patients with a CD4 count < 100 cells/µL as this has been demonstrated to improve survival [20].

In a recent two-year prospective observational cohort study conducted by Sungkanuparph et al. [87] in Thailand, 302 HIV–infected patients who had a CD4 T-cell count < 100 cells/µL and negative serum cryptococcal antigen initiating antiretroviral therapy were enrolled. Five patients developed cryptococcosis on either arm (2.5% of the fluconazole group and 5% of the control group) and two patients died, one in each group. This demonstrates that in settings where ART is widely available and HIV-infected patients who have CD4 counts < 100 cells/µL are initiated on ART and are negative for serum cryptococcal antigen, the primary prophylaxis for cryptococcosis with fluconazole has no survival benefit and may not be necessary [87].

In developed countries such as Europe and the USA, with a wide availability of ART, the low burden of HIV-related cryptococcosis and very low prevalence of cryptococcal antigenaemia, primary antifungal prophylaxis for cryptococcosis is not routinely recommended in HIV-infected patients [49,88]. A recent study from Uganda already demonstrated a high frequency of antifungal/fluconazole resistant *Cryptococcus* species from clinical isolates, and this could be attributed to the use of these agents for in primary prophylaxis or primary therapy for cryptococcosis [89].

### 5.2. Secondary Prophylaxis

This is also known as maintenance or suppressive therapy. The relapse rate for cryptococcal meningitis in persons with AIDS is more than 50% within the first year [60,90]. It is firmly established that oral fluconazole, 200 mg/day, is the drug of choice for maintenance treatment, although the consensus of older opinion favours life-long maintenance treatment [78]. Secondary prophylaxis decreases the rate of relapse to less than 5% in HIV/AIDS patients [60].The IDSA recommends that after a minimum of 12 months of antifungal therapy during ART, the discontinuation of suppressive antifungal therapy can be considered in patients with a CD4 count > 100 cells/µL and an undetectable or very low HIV RNA level sustained for ≥3 months. However, the maintenance therapy should be reinstituted if the CD4 cell count decreases to <100 cells/µL [49].

## 6. Cryptococcal Antigen Screening

As mentioned before, HIV-infected individuals with CD4 counts < 100 cells/mm^3^ are at the highest risk for developing cryptococcal disease. *Cryptococcus* spp. shed their polysaccharide capsular antigens very early into the bloodstream during systemic dissemination i.e., between five and 234 (median 22) days before the development of clinical cryptococcal disease; a clinical phenomenon now described “asymptomatic antigenaemia” [31]. The understanding of this fascinating pathophysiology allows a “window” of opportunity for a subclinical cryptococcal disease to be diagnosed through routine screening of high risk patients. A recent study from Addis Ababa, Ethiopia recruiting consecutive adult HIV-infected patients from two public HIV clinics found an overall prevalence of cryptococcal antigenaemia of 8.4%; 11% in patients with a CD4 count < 100 cells/µL, 8.9% with CD4 100 to 150 cells/µL and 5.7% with CD4 range of 150–200 cell/µL. About 84% of patients with cryptococcal antigenaemia were receiving ART [28]. The clinical significance of a positive CrAg and the role of pre-emptive antifungal therapy in patients with CD4 > 200 cells/µL remains an enigma. Significant deficits in neurocognitive function in asymptomatic CrAg positive persons with advanced HIV/AIDS even without signs or sequelae of meningitis have also been identified. Pre-emptive fluconazole treatment and the initiation of ART improved neurocognitive function in this group of patients [91].

In 2012, Jarvis et al. [92] proposed a treatment algorithm for the management of patients with asymptomatic cryptococcal antigenaemia. Patients who screen negative for CrAg should be enrolled into routine HIV care as per national/WHO guidelines. Those who screen positive are referred for lumbar puncture for CSF analysis and blood culture to rule out cryptococcal meningitis or disseminated disease; if the results are positive, it is treated as symptomatic meningoencephalitis and/or disseminated disease. For patients with asymptomatic cryptococcal antigenaemia, “pre-emptive” antifungal (fluconazole 400 mg per day orally) therapy is initiated and continued until immune reconstitution [20,52]. Serum CrAg positivity is independently associated with the onset of new cryptococcal meningitis and mortality during the first year of follow-up and mortality is highly correlated with CrAg titre [65,93]. In screen positive patients, about a third have concurrent meningitis, usually asymptomatic. CrAg titres in the blood are higher in those with meningitis. A cut-off titre of 1:160 appears to separate those with and without meningitis [94]. One of the commercially available lateral flow cryptococcal antigen tests has a double concentration line, approximating to a titre of 1:160. As some patients are reluctant to have a lumbar puncture, this additional data can be used to treat the patient as if they have meningitis [95].

In 2015, South Africa established a national cryptococcal antigenaemia screening policy targeting at ART naïve HIV-infected patients with CD4+ T-cell counts < 100 cells/µL. Two screening strategies are included in their national guidelines: reflex screening, where a CrAg test is performed on remnant blood samples from CD4 testing; and provider-initiated screening, where providers order a CrAg test after a patient returns for CD4 test results. CrAg screening before the initiation of ART was shown to potentially reduce the incidence of cryptococcal meningitis and save lives. Reflex screening compared to provider-initiated screening had more survival benefits and cost-effectiveness by reducing additional costs per additional year of life saved [96,97]. In settings with a high HIV prevalence, including CrAg screening in the initial work-up for meningitis has been shown to be highly cost-effective [98]. The “*screen and treat*” strategy in ARV-naïve patients with CD4 counts < 100 cells/µL have been conducted in various settings, including South Africa, Uganda, Vietnam and Cambodia [21,99,100,101]. All studies have demonstrated the cost-effectiveness of this strategy, even in settings with a lower prevalence estimate of 2% [18].

## 7. Conclusions

**Figure 4:**
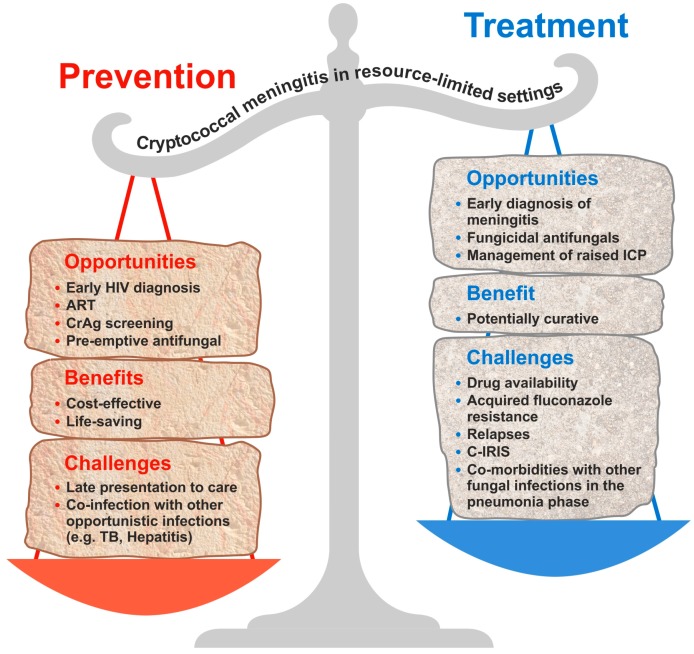
Opportunities, benefits and challenges of prevention and treatment of cryptococcosis in resource limited settings. C-IRIS. Cryptococcal-Immune reconstitution inflammatory syndrome. TB. Tuberculosis. ART. Anti-retroviral therapy. CrAg. Cryptococcal antigen. ICP. Intracranial pressure.

The burden of HIV-associated cryptococcal disease remains high, especially in resource-limited settings. We advocate for routine CrAg screening and pre-emptive antifungal therapy, especially in settings with a limited availability of ART, high levels of antiretroviral drug resistance, and a high burden of cryptococcal disease. This is a cost-effective public health intervention associated with reduced mortality by decreasing the incidence of cryptococcal meningitis among HIV-infected patients, especially in a population with a high prevalence (>3%) of cryptococcal antigenaemia. However, on the other hand, timely diagnosis and early treatment of an acute episode of cryptococcal meningitis and its associated complications (especially C-IRIS and raised ICP) is associated with better clinical outcomes. However, the time old adage still stands that “*prevention is better than cure*”. Figure 4 summarises our view of the existing opportunities, benefits and challenges of prevention and treatment of cryptococcosis in resource limited settings.

## Figures and Tables

**Table 1:** Cryptococcal meningitis burden and availability of the essential management package in resource-limited settings. A comparison of Uganda and Nigeria.

Parameter	Uganda	Nigeria
Population (2015)	40.1 million	181.2 million
Annual cases of cryptococcal meningitis	12,211 *	27,058 *
Annual mortality	10,120 *	24,972 *
Proportion of all AIDS deaths	23% *	14% *
Fluconazole	Available	Available
Amphotericin B	Available	Available (poor accessibility and unaffordable )
Flucytosine	Not available	Not available
Lumbar puncture	Routinely done	Not done (adults)
Manometry	Routinely done	Not done
Cryptococcal lateral flow assays	Available	Not available
National cryptococcal screening and treatment program	Available	Not available
Expert physicians in cryptococcal disease management	>5	2 or 3
Clinical trials on cryptococcal meningitis	>5	None
Centre of excellence	Yes	No

* Data from Rajasingham et al. [6].
